# Well-Defined Polypropylene/Polypropylene-Grafted Silica Nanocomposites: Roles of Number and Molecular Weight of Grafted Chains on Mechanistic Reinforcement

**DOI:** 10.3390/polym8080300

**Published:** 2016-08-12

**Authors:** Masahito Toyonaga, Patchanee Chammingkwan, Minoru Terano, Toshiaki Taniike

**Affiliations:** School of Materials Science, Japan Advanced Institute of Science and Technology, 1-1 Asahidai, Nomi, Ishikawa 923-1292, Japan; toyonaga@tei-c.com (M.T.); chamming@jaist.ac.jp (P.C.); terano@jaist.ac.jp (M.T.)

**Keywords:** polypropylene, nanocomposite, grafting technique, polymer-grafted nanofiller, co-crystallization, physical cross-linkage, mechanical properties, tensile test, materials design

## Abstract

Grafting terminally functionalized polypropylene (PP) to nanofillers provides well-defined PP-based nanocomposites plausibly featured with a physical cross-linkage structure. In this paper, a series of PP-grafted silica nanoparticles (PP-*g*-SiO_2_) were synthesized by varying the number of grafted chains per silica particle, and influences of the number and the molecular weight of grafted chains were studied on physical properties of PP/PP-*g*-SiO_2_ nanocomposites. We found that only 20–30 chain/particle was sufficient to exploit benefits of the PP grafting for the nanoparticle dispersion, the nucleation, and the Young’s modulus. Meanwhile, the yield strength was sensitive to both of the number and the molecular weight of grafted PP: Grafting longer chains at a higher density led to greater reinforcement.

## 1. Introduction

Growing production and applications of polymer materials have owed much to a variety of compounding technologies. In particular, polymer nanocomposites are one of the most promising materials, where the inclusion of nanosized fillers in polymer matrices can improve (or endow) a variety of properties including mechanical [1,2,3,4], thermal [5,6], flame retardant [7,8], electric [9,10], electromagnetic [11,12], optical [13,14], barrier [15,16], and other properties [17,18,19,20]. Numerous researches have been implemented for each different combination of polymer and nanofillers. Among these, polypropylene (PP)-based nanocomposites have attracted particular attention, owing to the hugest market of PP and a multitude of potential applications [21]. 

The key issue for formulating PP-based nanocomposites is how to overcome poor compatibility and weak interfacial bonding arising from the chemical inertness of PP. The former is relevant to the dispersion of nanofillers in PP matrices, while the latter is important for load transfer to nanofillers [22] and durability against failures [23,24]. The compatibility problem is relatively easily solved through the addition of a compatibilizer such as maleic anhydride-grafted PP and organic modification of filler surfaces typically using silane coupling agents. Even without dispersants, in-situ methods can facilitate forcibly dispersed nanofillers, which include propylene polymerization in the presence of catalyst-bearing nanofillers [25,26] and synthesis of nanoparticles from molecular precursors in molten PP [27,28]. 

For strengthening the interfacial bonding, the most effective strategy is the polymer grafting. Polymer chains grafted onto filler surfaces not only aid the compatibilization of nanofillers but also entangle/interdiffuse with matrix polymer [29,30,31,32,33,34,35]. It is known that polymer chains grafted at their chain end offer much better interfacial reinforcement as compared to those grafted at their side chains [36]. This is because side-functionalized polymer chains wrap nanofillers and form a soft interfacial layer between PP and nanofillers, which hampers the hardness of nanofillers. 

**Figure 1:**
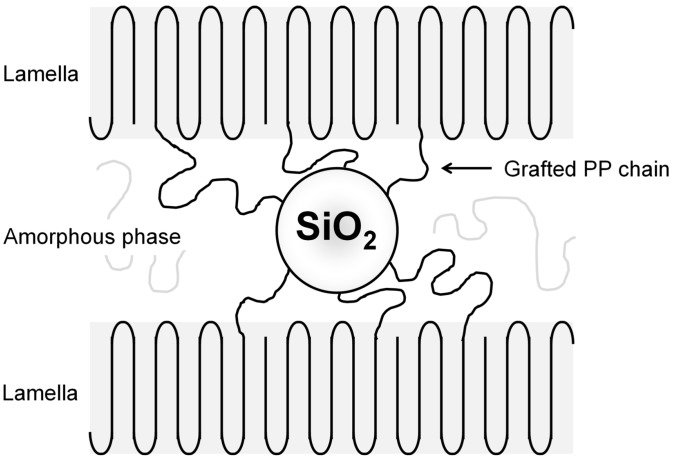
Schematic illustration of a physical cross-linkage structure for polypropylene (PP)/PP-grafted SiO_2_ (PP-*g*-SiO_2_) nanocomposites.

While many kinds of polymer have been grafted to nanofillers for PP nanocomposites [37,38,39,40,41,42], our recent efforts are regarded as one of a few successful examples that have employed terminally functionalized PP chains grafted to nanofillers [43,44]. In detail, terminally hydroxylated isotactic PP (PP-*t*-OH) with well-defined primary structures was grafted to silica (SiO_2_) nanoparticles, and thus obtained PP-grafted SiO_2_ (PP-*g*-SiO_2_) nanoparticles were melt mixed with PP. We found several positive consequences of the PP grafting on boosting physical properties of resultant nanocomposites: Good dispersion up to 10 wt %, at maximum 4 times faster crystallization compared with pristine PP, and approximately +30% improvement in the Young’s modulus and yield strength. Especially, the +30% improvement of the yield strength was unexpectedly large as for spherical fillers of low aspect ratios. Terminally grafted PP chains worked as crystallization nuclei and co-crystallized with matrix PP. It was envisaged that PP-*g*-SiO_2_ would bridge neighboring lamellae through the co-crystallization and thus formed physical cross-linkage structure would be responsible for the unexpected reinforcement (Figure 1). In line with the above expectation, the nanocomposites completely lost the anomalous reinforcement when the lamellae were dissolved in a molten state. 

Based on our previous results [44], the maximum tensile reinforcement was achieved over a critical molecular weight of 1.2 × 10^4^ for PP-*t*-OH grafted to SiO_2_ nanoparticles. In this report, we have systematically varied the number of grafted PP chains per SiO_2_ nanoparticle, and attempted to separately study impacts of the molecular weight and the number of the grafted chains on physical properties of PP/PP-*g*-SiO_2_ nanocomposites. As long as we recognize, such a comprehensive study on the design of polymer-grafted nanoparticles for polymer nanocomposites has been limited to polystyrene that can be relatively easily controlled [32,45,46], and rarely performed for PP that necessitates end functionalization during catalyzed olefin polymerization. 

## 2. Materials and Methods

### 2.1. Materials

PP pellets (*M*_n_ = 4.6 × 10^4^, *M*_w_/*M*_n_ = 5.65, *mmmm* = 98 mol %) were donated from Japan Polypropylene Corporation. Propylene gas of research grade (Japan Polypropylene Corporation, Tokyo, Japan) was used as delivered. *rac*-Ethylenebis(1-indenyl)zirconium dichloride (EBIZrCl_2_, metallocene catalyst) was purchased from Kanto Chemical Co., Inc. (Tokyo, Japan). Triethylaluminum (TEA, chain transfer agent) and modified methylaluminoxane (MMAO, activator) were donated by Tosoh Finechem Corporation (Shunan, Japan). Toluene and *n*-tetradecane were dried over molecular sieve 4A followed by N_2_ bubbling. SiO_2_ nanoparticles with an average diameter of 26 nm and a surface area of 110 m^2^/g (Kanto Chemical Co., Inc.) were dried at 160 °C for 4 h prior to the grafting reaction.

### 2.2. Synthesis of Hydroxylated Isotactic Polypropylene (PP-t-OH)

PP-*t*-OH was prepared according to literature [44]. Briefly, 300 mL of dried toluene, 4.5 mmol of MMAO, 1.5 μmol of EBIZrCl_2_, and 9.0 mmol of TEA were charged in a stirred flask maintained at 0 °C. Propylene polymerization was conducted at 0 °C by supplying 1 atm of propylene for 1 h. The activator (MMAO) alkylates the catalyst precursor (EBIZrCl_2_) and extracts an alkyl anion to form cationic active species (EBIZrR^+^). To the formed active species, propylene monomer is coordinated and inserted to cause the chain growth. The chain growth is terminated through chain transfer reactions such as β-hydride elimination, chain transfer to monomer, and that to alkylaluminum. The former two chain transfer pathways bear terminally unsaturated PP, while the last one produces PP-*t*-Al. TEA works as a chain transfer agent to form PP-*t*-Al, whose concentration controls the molecular weight of the produced PP via chain transfer frequency. The low polymerization temperature was important to suppress the undesired chain transfer reactions. The polymerization product was reacted with 25 mL of 35 mol % aqueous H_2_O_2_ under oxygen atmosphere at 0 °C for 1 h, and subsequently hydrolyzed with methanol for 30 min. These post-polymerization treatments convert (a majority of) PP-*t*-Al into PP-*t*-O-O-Al and eventually into PP-*t*-OH [47,48]. The product was purified by repetitive washing with acidic ethanol and subsequent vacuum drying prior to characterization. 

The primary structure of PP-*t*-OH was characterized mainly based on ^13^C{^1^H} NMR (Bruker 400 MHz operating at 100 MHz) at 120 °C using hexachloro-1,3-butadiene as a diluent and 1,1,2,2-tetrachloroethane-*d*_2_ as an internal lock and reference. The analytical method for the obtained spectra was described elsewhere [47]. Briefly, the isotacticity of PP-*t*-OH was determined as 85 mol % in *mmmm*, which was less than 98 mol %, but sufficiently isotactic for co-crystallization with the matrix PP (see Figure S2 in Reference [44]). Terminal groups of PP-*t*-OH consisted of methyl as chain heads and hydroxyl as chain ends. Their molar ratio derived the end functionalization ratio of 73 mol % (i.e., 27 mol % of chains were not functionalized). The molecular weight (*M*_n_) of PP-*t*-OH was evaluated as 1.2 × 10^4^ based on the molar ratio between main chain carbons and terminal carbons. Owing to the narrow molecular weight distribution of metalloncene-catalyzed PP-*t*-OH, the *M*_n_ values were quantitatively comparable between ^13^C NMR and gel permeation chromatography [44].

### 2.3. Synthesis of Polypropylene-Grafted SiO_2_ (PP-*g*-SiO_2_)

PP-*t*-OH was grafted onto SiO_2_ nanoparticles via condensation between the terminal hydroxyl group of PP-*t*-OH and surface silanol groups of SiO_2_. A specified amount of PP-*t*-OH (0.1–4.0 g) and 0.5 g of SiO_2_ nanoparticles were reacted in tetradecane containing 0.1 mol/L of 6-di-*tert*-butyl-*p*-cresol at 200 °C for 6 h under N_2_ and vigorous stirring. Unreacted polymer was carefully removed with repetitive hot filtration in *o*-dichlorobenzene at 140 °C. The resultant particles were washed with methanol, and dried in vacuo at 60 °C for 6 h. It must be noted that PP-*t*-OH retained the capability of co-crystallization with the matrix PP even after being grafted (again see Figure S2 in Reference [44]). 

### 2.4. Synthesis of PP/PP-g-SiO_2_ Nanocomposites

PP-based nanocomposites were prepared by a two-roll mixer at 20 rpm. 10 g of the PP pellets was kneaded at 185 °C for 5 min, followed by the addition of a specified amount of unmodified SiO_2_ or PP-*g*-SiO_2_ nanoparticles (0, 1, 3, 5 wt %). The mixture was kneaded at 185 °C for additional 10 min. Sample films with the thickness of 200 μm were prepared by hot press at 230 °C and 20 MPa, followed by quenching at 100 °C for 5 min. 

### 2.5. Analyses

Thermogravimetric analysis (TGA, METTLER TOLEDO TG50, Columbus, OH, USA) was employed to measure the amount of grafted polymer. The temperature was kept at 200 °C for 30 min to remove physisorbed water, and then increased up to 650 °C at 20 °C/min. The grafted amount was estimated from the difference in the weight loss between PP-*g*-SiO_2_ and unmodified SiO_2_ in the range of 200–650 °C. The weight of grafted chains was converted into the chain number using *M*_n_, while the number of nanoparticles in the unit gram of SiO_2_ was estimated from the weight of one nanoparticle with the sphere diameter of 26 nm and the density of 2.2 g/cm^3^. Thus, the grafted amount was finally expressed in the unit of the chain number per particle. The dispersion of SiO_2_ nanoparticles was observed by a transmission electron microscope (TEM, Hitachi H-7100, Tokyo, Japan), using specimens with the thickness of 100 nm, which were prepared by an ultramicrotome (Reichert Ultracut S with a FC-S cryoattachement, Leica Microsystems, Wetzlar, Germany). The crystallization behavior of PP was examined using differential scanning calorimetry (DSC, METTLER TOLEDO DSC 822, Columbus, OH, USA). A sample was heated from room temperature to 200 °C at 20 °C/min, then kept at 200 °C for 5 min to eliminate the thermal history, and finally cooled down to 128 °C at 20 °C/min for the isothermal crystallization. The endotherm in the first heat cycle was employed to aquire the melting point (*T*_m_), the crystallinity (*X*_c_), and the distribution of lamellar thickness (*l*_c_) [49,50,51,52]. The isothermal crystallization rate (denoted as *t*_1/2_^−1^) was defined as an inverse of the half time of the crystallization at 128 °C. Wide-angle X-ray diffraction (WAXD) measurements were performed in a reflection mode at room temperature with Cu Kα radiation operating at 40 kV and 30 mA. The scanning rate was 1°/min over 2θ of 10°–30°. Tensile properties were measured by a tensile tester (Abe Dat-100, Kanazawa, Japan). A stress-strain curve was acquired at room temperature and at a crosshead speed of 1.0 mm/min. Tensile properties were calculated as the average of 5 measurements. 

## 3. Results and Discussion

A list of PP-*g*-SiO_2_ samples used in this study is summarized in Table 1. A part of the samples was taken from Reference [44] in order to separately discuss impacts of the amount and length of grafted PP chains on physical properties of resultant nanocomposites. When 667 μmol of PP-*t*-OH was reacted with one gram of SiO_2_ nanoparticles in the grafting reaction, the amount of grafted PP chains always fell in the range of 10–11 wt % irrespective of the molecular weight of PP-*t*-OH (except the shortest PP-*t*-OH with *M*_n_ = 5.8 × 10^3^). This result was interpreted as follows: Grafted chains form polymer layer on SiO_2_ surfaces and make it increasingly difficult for ungraftd chains to reach bare surfaces. Since the thickness of the polymer layer is determined by the grafted amount rather than the molecular weight, the graftable amount became independent of the molecular weight, and thus the number of grafted chains per SiO_2_ nanoparticle decreases anti-proportionally to the molecular weight of PP-*t*-OH. By changing the ratio between the two reactants, PP-*t*-OH and SiO_2_ while fixing *M*_n_ of PP-*t*-OH at 1.2 × 10^4^, the grafted amount was controlled from 2.2 to 11.4 wt %, corresponding to 21 and 120 chain number per particle, respectively. The grafted amount increased in a convergent manner against the addition amount of PP-*t*-OH, in agreement with the above-explained self-limiting mechanism.

**Figure 2:**
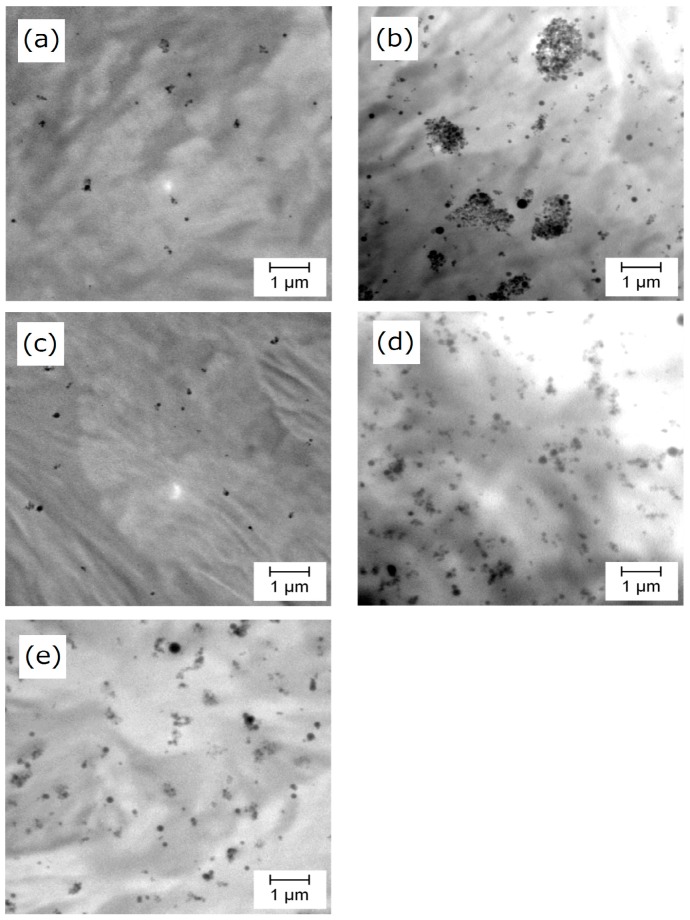
Transmission electron microscope (TEM) images for (**a**) PP/SiO_2_ (1 wt %); (**b**) PP/SiO_2_ (5 wt %); (**c**) PP/120PP120-*g*-SiO_2_ (1 wt %); (**d**) PP/120PP120-*g*-SiO_2_ (5 wt %), and (**e**) PP/21PP120-*g*-SiO_2_ (5 wt %).

Nanocomposites were prepared by melt mixing 1–5 wt % of PP-*g*-SiO_2_. Figure 2 shows TEM micrographs for selected samples to evaluate the dispersion of the nanoparticles in the matrix. Unmodified SiO_2_ nanoparticles were relatively uniformly dispersed at 1.0 wt % (Figure 2a), but formed micron-sized compact aggregates at 5.0 wt % (Figure 2b). In contrast to unmodified SiO_2_, PP-*g*-SiO_2_ showed uniform dispersion at 1–5 wt % (Figure 2c,d), and the dispersion was not deteriorated even when the chain number per particle was reduced to 21 (Figure 2d,e). The chain number per particle of 21 corresponds to 0.01 chain/nm^2^, and a PP chain with *M*_w_ = 2.4 × 10^4^ (calculated from *M*_n_ = 1.2 × 10^4^ based on *M*_w_/*M*_n_ = 2) occupies 122 nm^2^/chain at the random-coil state [53,54]. Multiplying 0.01 chain/nm^2^ by 122 nm^2^/chain leads to 121% coverage of SiO_2_ surfaces. In this way, it is estimated that SiO_2_ surfaces were fully covered by grafted PP even at 21 chain/particle, and the surface energy of PP-*g*-SiO_2_ was believed to be similar in the range of 21–120 chain/particle, which plausibly explains uniform dispersion at 21 chain/particle. The coverage for 120PP120-*g*-SiO_2_ is similarly estimated as 692%. Even with similar surface energies, the state of grafted chains must change from random-coil to brush-like as the coverage increases [45,46,54]. The coverage for 140PP58-*g*-SiO_2_, 160PP87-*g*-SiO_2_, 70PP180-*g*-SiO_2_, 40PP330-*g*-SiO_2_, and 30PP460-*g*-SiO_2_ was respectively estimated as 358%, 644%, 636%, 716% and 779%. Thus, it was believed that SiO_2_ nanoparticles were (almost) fully covered by grafted PP chains, offering similarly uniform dispersion.

**Figure 3:**
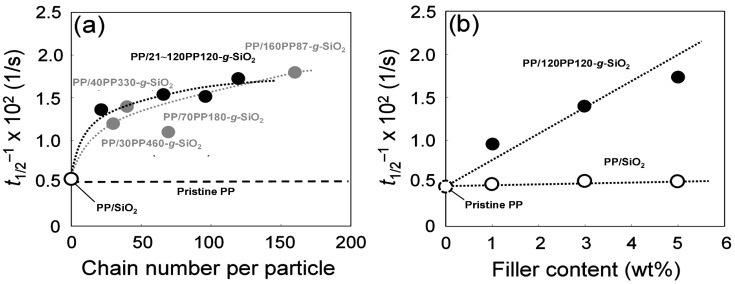
Variation of the crystallization rate at 128 °C (**a**) along the chain number per particle at the fixed filler content (5.0 wt %), and (**b**) along the filler content at the fixed chain number per particle (120 chain/particle): (○,●,●) PP/SiO_2_, PP/*xxx*PP120-*g*-SiO_2_, and PP/*xxx*PP*yyy*-*g*-SiO_2_. The data for PP/*xxx*PP*yyy*-*g*-SiO_2_ is taken from Reference [44].

DSC results are summarized in Table 2, again referring to our previous data [44]. The addition of unmodified SiO_2_ and PP-*g*-SiO_2_ hardly affected the crystallinity, and the melting temperature seemed to be slightly lowered by 1–2 °C. On the other hand, the PP grafting endows a nucleation ability to SiO_2_ nanoparticles, leading to marked enhancement in the crystallization rate. Figure 3a plots the crystallization rate along the chain number per particle at the filler content of 5.0 wt %. For comparison, our previous data for PP/PP-*g*-SiO_2_ nanocomposites [44] are also plotted. These nanocomposites contained 5.0 wt % of PP-*g*-SiO_2_ with different molecular weights of the grafted chains, where the chain number per particle decreased roughly anti-proportionally to the molecular weight. It was found that the crystallization rate followed similar correlation curves for the chain number per particle, irrespective of the way of its variation (i.e., either through the grafted amount or through the molecular weight). Most of the enhancement was achieved only with 20–30 chain/particle, and additional grafting resulted in small increments even though each grafted chain was regarded as a crystallization nucleus. This fact meant that the number of crystallites formable per particle is limited plausibly due to a steric reason. In Figure 3b, the crystallization rate was found to increase in proportion to the PP-*g*-SiO_2_ content. This fact indicated that the nanoparticles as crystallization nuclei dispersed similarly uniformly in the range of 1–5 wt %.

**Figure 4:**
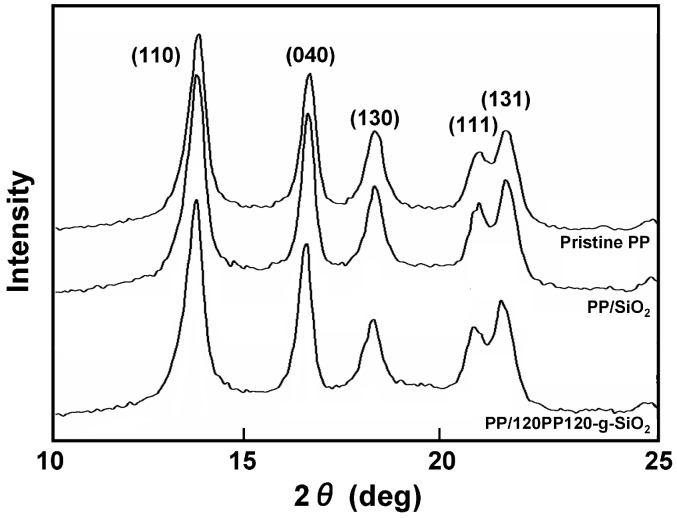
Wide-angle X-ray diffraction (WAXD) patterns.

**Figure 5:**
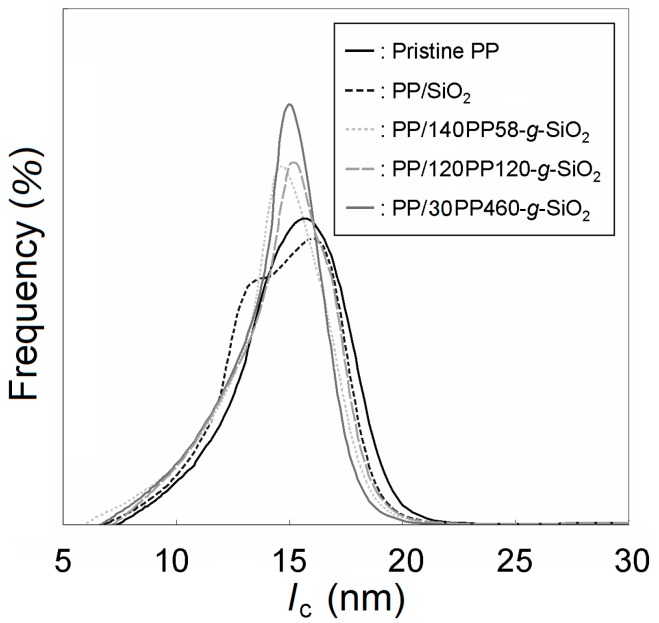
Lamellar thickness distribution acquired based on the DSC endotherm in the first heating.

As described above, the inclusion of PP-*g*-SiO_2_ nanoparticles did not affect the melting point and crystallinity of the matrix PP, while their nucleation ability might suggest potential influences on higher-order structures of the matrix. For instance, the formation of trans crystals on filler surfaces can be important in indentifying the origin of mechanical reinforcement [55]. Here, results of additional structural characterization are reported for PP/120PP120-*g*-SiO_2_ at 5.0 wt %. Figure 4 compares WAXD patterns among pristine PP, PP/unmodified SiO_2_, and PP/120PP120-*g*-SiO_2_. All the samples showed typical characteristics of the α-form, without any noticeable changes in the peak intensity ratio and the peak widths. Figure 5 displays the distribution of lamellar thickness (*l*_c_) that was derived from the DSC endotherm in the first heating. For pristine PP, the lamellar thickness ranges from 8 to 21 nm with the peak top located at 16 nm. The inclusion of unmodified SiO_2_ nanoparticles added a shoulder at about 13 nm to the original distribution. As observed in Figure 2b, the dispersion of unmodified SiO_2_ nanoparticles was highly non-uniform. A local high concentration of nanoparticles (agglomeration) confined the growth of lamellae [56,57]. In the case of PP-*g*-SiO_2_, the distribution was downward shifted with the new peak top located at 15 nm. The dimensional reduction of the higher-order structure was reasonable as PP-*g*-SiO_2_ nanoparticles offered the crystallization nuclei. Stern et al. reported that the tensile stress of pristine PP develops proportionally to the lamellar thickness [58]. Considering all of these facts, mechanical reinforcement in PP/PP-*g*-SiO_2_ nanocomposites was hardly attributed to the alternation of higher-order structures by the addition of PP-*g*-SiO_2_. It must be noted that even though the addition of short alkyl chain-modified SiO_2_ nanoparticles causes similar consequences to higher-order structures of PP, the extent of reinforcement is far less than that brought by PP-*g*-SiO_2_ [43]. 

**Figure 6:**
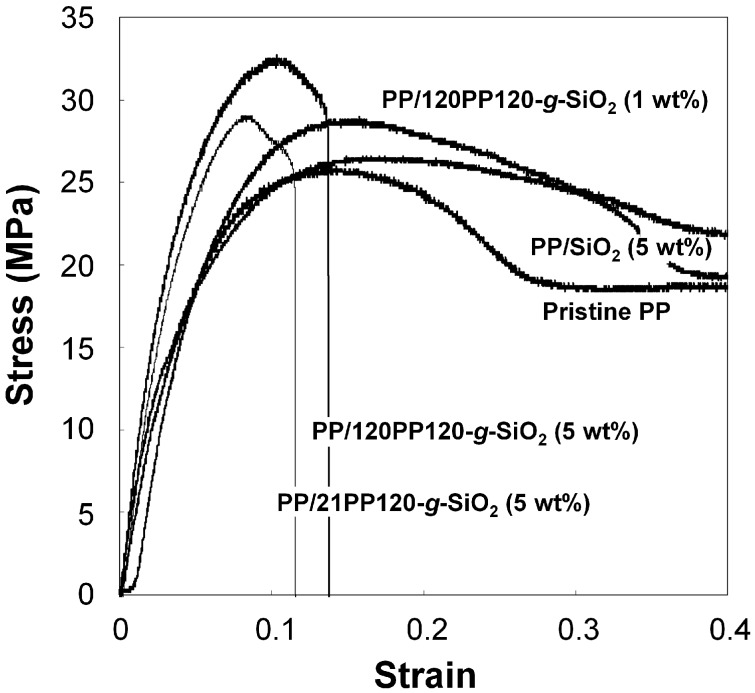
Stress–strain curves.

Uniaxial tensile properties of the prepared nanocomposites were investigated. Typical stress-strain curves are displayed in Figure 6. The addition of unmodified SiO_2_ hardly affected the stress development at a lower strain, leading to little improvement in the Young’s modulus and yield stress. The addition of PP-*g*-SiO_2_ markedly enhanced these properties through the improved dispersion and interfacial bonding, which necessarily caused great reduction in the ductility at 5.0 wt %.

**Figure 7:**
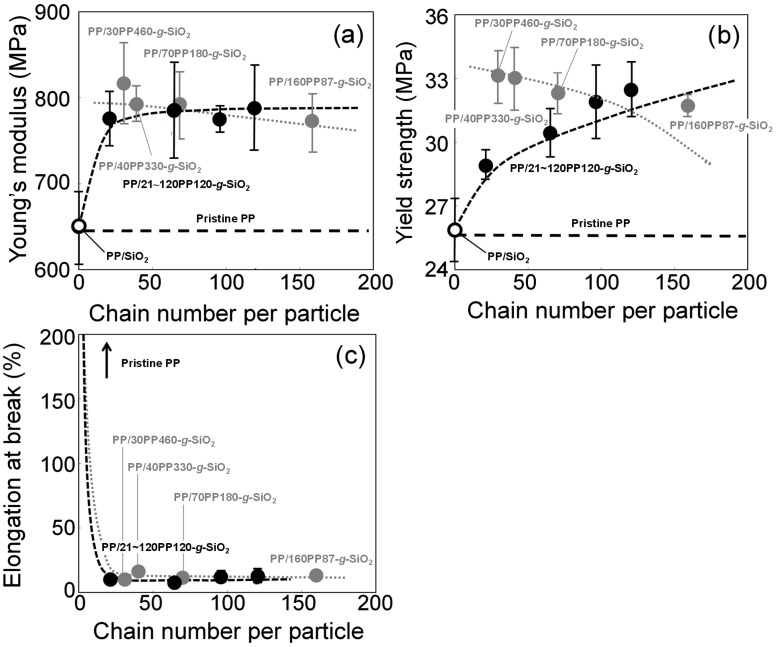
Tensile properties as a function of the chain number per particle: (**a**) Young’s modulus; (**b**) yield strength; and (**c**) elongation at break. The symbols (○,●,●) correspond to PP/SiO_2_, PP/*xxx*PP120-*g*-SiO_2_, and PP/*xxx*PP*yyy*-*g*-SiO_2_, respectively. The data for PP/*xxx*PP*yyy*-*g*-SiO_2_ is taken from Reference [44].

The Young’s modulus, yield strength and elongation at break were calculated as average values among 5 specimens for each nanocomposite. Figure 7 plots the tensile properties of the nanocomposites at 5.0 wt % against the grafted chain number per particle. Again, our previous data with varied molecular weights of grafted chains are included for the sake of comparison. It was expected that not only the chain number per particle but also the molecular weight of grafted chains might contribute to the enhancement of the Young’s modulus and yield strength, as both the factors enrich interfacial bonding and physical cross-linkage. For the Young’s modulus, the reinforcement almost reached the maximum (around +30% compared with that of pristine PP) at 21 chain/particle, and further grafting hardly improved it. It was also observed that the molecular weight of the grafted chains hardly affected the extent of the reinforcement. García et al. succeeded to disperse colloidal SiO_2_ nanoparticles in PP without any chemical modification or additives, and achieved +30% reinforcement of the Young’s modulus compared with pristine PP [59]. This fact suggested that uniform dispersion of hard nanoparticles is sufficient to improve the Young’s modulus and the interfacial bonding is unnecessary, which is in line with our previous findings that the modulus was insensitive to the details of grafted chains [44]. 

Differently from the Young’s modulus, the yield strength was greatly dependent on the chain number per particle (Figure 7b). While the increment from 0 to 21 chain/particle was the greatest (since the dispersion improvement also contributed), further grafting led to monotonous reinforcement of the yield strength and finally reached +27% reinforcement at 120 chain/particle compared with pristine PP. The increment in the molecular weight of grafted chains also led to enhanced reinforcement in spite of the reduction in the chain number per particle. Thus, it was clear that both the chain number and the molecular weight of grafted chains are critical for the improvement of the yield strength. The maximum reinforcement over pristine PP has reached +30% which was unexpectedly high for the inclusion of spherical nanoparticles. Such anomalous reinforcement in the yield strength had been attributed to a physical cross-linkage structure (Figure 1), in which PP-*g*-SiO_2_ bridges matrix lamellae with a fraction of grafted chains involved in the lamellae through co-crystallization [44]. Combination between the great reinforcement in the solid state and scarce improvement of shear modulus in the molten state had supported the above hypothesis in an analogous fashion to thermoelastomer [60]. In such a viewpoint, it was believed that the observed sensitivity of the yield strength on the density and molecular weight of grafted chains was relevant to the extent of the physical cross-linkage, i.e., a chance of the cross-linkage must enhance as the density and molecular weight of grafted chains increase. The elongation at break was dramatically reduced in the presence of grafted chains, plausibly due to the restricted deformation via a physical cross-linkage structure. 

Influences of the filler content were also examined. All of the properties varied nearly proportionally to the filler content, which again suggested that the dispersion of PP-*g*-SiO_2_ was unaltered in the range of 1–5 wt %, and that the tensile reinforcement of the matrix was originated from PP-*g*-SiO_2_ nanoparticles. 

In summary, the PP grafting caused both reinforcement and embrittlement of the nanocomposites. Interface design through the chain number and molecular weight of the grafted chains was critical for the yield strength, but not for the other properties. Except the ductility, the best nanocomposite with a physical cross-linkage structure was obtained at the highest filler content (as long as good dispersion is retained), and with the greatest chain number per particle and the highest molecular weight of the grafted chains. 

## 4. Conclusions

PP/PP-*g*-SiO_2_ nanocomposites featured with a physical cross-linkage structure offer a multitude of advantages such as uniform dispersion of nanoparticles, accelerated crystallization, and great tensile reinforcement. Starting from PP-*t*-OH with well-defined primary structures, a series of PP-*g*-SiO_2_ nanoparticles were synthesized, and materials design for PP-*g*-SiO_2_ was comprehensively studied on physical properties of PP/PP-*g*-SiO_2_ nanocomposites. The findings are summarized below.

1. As long as filler surfaces are mostly covered by grafted chains, the number of grafted chains per particle is not important for improving the dispersion of nanoparticles. 21 chain/particle (corresponding to 120% coverage) was sufficient in our system.
2. The presence of grafted chains is essential to endow a nucleating ability to SiO_2_ nanoparticles. The density of dispersed PP-*g*-SiO_2_ nanoparticles in PP is important to enhance the crystallization rate, rather than the number of grafted chains.
3. The Young’s modulus is not sensitive to the interfacial bonding as long as nanoparticles are well dispersed. Meanwhile, both the number and the molecular weight of grafted chains are crucial for improving the yield strength.

In the end, the significance of the synthetic controls must be noted in realizing such a comprehensive study with reliable conclusions on PP-based nanocomposites. Especially, functionalization in metallocene-catalyzed olefin polymerization and complete removal of ungrafted chains by repetitive washing were essential for the well-defined nature of the study. On the other hand, these processes were far inefficient to be industrially viable. It is expected that the obtained conclusions would be embodied within improved synthetic strategies. 

## Figures and Tables

**Table 1:** List of PP-*g*-SiO_2_ samples used in this study and their characteristics.

Sample ^a^	PP-*t*-OH amount (μmol per gram of SiO_2_) ^b^	Grafted amount (wt %) ^c^	Chain number per particle ^d^
21PP120-*g*-SiO_2_	16.7	2.2	21
65PP120-*g*-SiO_2_	83.3	6.3	65
96PP120-*g*-SiO_2_	333	9.1	96
120PP120-*g*-SiO_2_	667	11.4	120
140PP58-*g*-SiO_2_^e^	667	6.4	140
160PP87-*g*-SiO_2_^e^	667	10.7	160
70PP180-*g*-SiO_2_^e^	667	9.6	70
40PP330-*g*-SiO_2_^e^	667	10.4	40
30PP460-*g*-SiO_2_^e^	667	10.9	30

^a^ Nanoparticles are termed as *xxx*PP*yyy*-*g*-SiO_2_ with *xxx* as the grafted chain number per particle and *yyy* as *M*_n_/100; ^b^ The amount of PP-*t*-OH used for the grafting reaction; ^c^ Determined with TGA; ^d^ The chain number per particle was estimated using *M*_n_ of PP-*t*-OH, the measured grafted amount, and the number of SiO_2_ nanoparticles per gram. The last parameter was deduced from the specific surface area and the diameter of SiO_2_ nanoparticles (110 m^2^/g and 26 nm, respectively); ^e^ Obtained from Reference [44].

**Table 2:** List of PP-*g*-SiO_2_ samples used in this study and their characteristics.

Sample	*T*_m_ (°C) ^a^	*X*_c_ (%) ^a^	*t*_1/2_^−1^ × 10^2^ (1/s) ^b^
Pristine PP	163	50	0.46
PP/SiO_2_ (1 wt %)	160	49	0.48
PP/SiO_2_ (3 wt %)	161	49	0.52
PP/SiO_2_ (5 wt %)	162	47	0.51
PP/140PP58-*g*-SiO_2_ (5 wt %) ^c^	159	50	2.1
PP/140PP87-*g*-SiO_2_ (5 wt %) ^c^	162	51	1.8
PP/21PP120-*g*-SiO_2_ (5 wt %)	163	51	1.3
PP/65PP120-*g*-SiO_2_ (5 wt %)	162	51	1.5
PP/96PP120-*g*-SiO_2_ (5 wt %)	161	52	1.4
PP/120PP120-*g*-SiO_2_ (1 wt %)	161	50	0.90
PP/120PP120-*g*-SiO_2_ (3 wt %)	162	49	1.4
PP/120PP120-*g*-SiO_2_ (5 wt %)	160	51	1.7
PP/70PP180-*g*-SiO_2_ (5 wt %) ^c^	163	48	1.1
PP/40PP330-*g*-SiO_2_ (5 wt %) ^c^	162	51	1.4
PP/30PP460-*g*-SiO_2_ (5 wt %) ^c^	161	53	1.2

^a^ Obtained from the first heat cycle; ^b^ Isothermal crystallization at 128 °C; ^c^ Obtained from Reference [44].
